# Engineering Ligand and Receptor Pairs with LIPSTIC to Track Cell-Cell Interactions

**DOI:** 10.1002/cpz1.311

**Published:** 2021-12-01

**Authors:** Dafne Alberti, Michelle Guarniero, Agnieszka K. Maciola, Enrico Dotta, Giulia Pasqual

**Affiliations:** 1 Laboratory of Synthetic Immunology, Department of Surgery, Oncology and Gastroenterology, University of Padova, Padova, Italy; 2 Veneto Institute of Oncology IOV-IRCCS, Padua, Italy

**Keywords:** cell-cell interactions, enzymatic labeling, immune interactions, LIPSTIC

## Abstract

Interactions between different cell types are critical for a plethora of biological processes, such as the immune response. We recently developed a novel technology, called LIPSTIC (labeling of immune partnership by SorTagging intercellular contacts), that allows for identifying cells undergoing specific interactions thanks to an enzymatic labeling reaction. Our work demonstrated the use of this technology to monitor interactions between immune cells, both in vitro and in vivo, by the genetic engineering of CD40 and CD40L, an essential costimulatory axis between antigen-presenting cells and T cells. Here we describe protocols to design novel LIPSTIC-engineered ligand and receptor pairs, clone constructs into retroviral expression vector, perform their initial validation, and use them to measure interactions ex vivo. This information will be useful to investigators interested in exploiting the LIPSTIC technology to track their favorite immune interaction.

**Basic Protocol 1:** Design of LIPSTIC-engineered ligand and receptor pairs

**Basic Protocol 2:** Cloning of LIPSTIC-engineered ligand and receptor pairs

**Basic Protocol 3:** Validation of LIPSTIC-engineered ligand and receptor pairs in 293T cells

**Basic Protocol 4:** Measuring interaction with LIPSTIC in immune cells ex vivo

## Introduction

Interactions between different cells play crucial roles in many biological processes, including fetal development, neural signaling, and tissue organization. Cell-cell interactions are of tremendous importance in the context of the immune system, since the majority of events that mediate an immune response do indeed rely on direct cell-cell contact. The activation of naive T cells by interaction with dendritic cells, the cytotoxic activity of effector CD8^+^ T cells toward their target, or the activation of tissue macrophages by CD4^+^ T cells represent just few examples of how the immune response is directed by interactions between distinct cell types.

**Figure 1:**
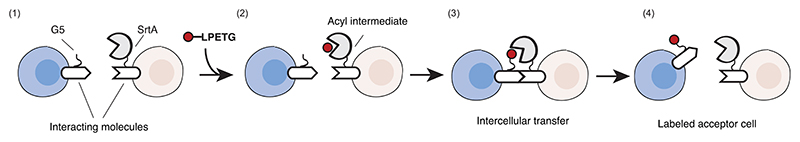
Schematic representation of the LIPSTIC approach to achieve intercellular enzymatic labeling. (1) Interacting molecules are genetically engineered to express either a five glycine (G5) tag or the Sortase A (SrtA) enzyme at their extracellular portion. (2) Upon addition of a biotinylated or fluorescently labeled SrtA substrate (i.e., a short peptide containing the LPETG sequence), SrtA forms a covalent acyl intermediate with the substrate. (3) Upon ligand-receptor interaction, SrtA catalyzes the covalent transfer of the labeled substrate to the G5-tagged molecule. (4) When the interaction is terminated, the cell participating in the interaction can be identified thanks to the presence of the labeled substrate on its surface.

Traditionally interactions have been observed by live-cell imaging. Nevertheless, microscopy-based approaches do not allow for retrieving cells participating in a given interaction of interest, and therefore the molecular and phenotypic changes occurring in interacting cells cannot be investigated. To overcome this limitation, we recently developed a novel technology called LIPSTIC (labeling of immune partnership by SorTagging intercellular contacts) that allows for enzymatically labeling cells undergoing interactions so that they can be easily retrieved for downstream analysis (Chudnovskiy, Pasqual, & Victora, 2019; Pasqual et al., 2018). LIPSTIC is based on Sortase A (SrtA; Jacobitz, Kattke, Wereszczynski, & Clubb, 2017), a transpeptidase capable of transferring a labeled substrate to an N-terminal five glycine (G5) tag. In LIPSTIC, a ligand and receptor are engineered to express at their extracellular portion either SrtA or the G5 tag. In the presence of the substrate, we initially observe the formation of a covalent intermediate between SrtA and the substrate itself; then, upon ligand-receptor interaction, SrtA catalyzes the ligation of the labeled substrate to the G5-tagged molecule, allowing the identification of the cell participating in the interaction of interest even when the interaction is terminated (Fig. 1).

We originally developed this technology by engineering CD40 and CD40L molecules to track interactions between antigen-presenting cells and T cells and validated this technology in vitro, ex vivo, and in vivo. Here we describe protocols to design, clone, and validate novel LIPSTIC-engineered ligand and receptor pairs and also provide a general protocol to measure interactions between immune cells ex vivo. Collectively these protocols have the scope of enabling scientists to take advantage of the LIPSTIC approach for the study of their immune interactions of interest.

*NOTE:* Retroviral vectors must be handled in Biosafety Level 2 facilities. Follow all appropriate guidelines and local regulations for the use and handling of retroviral vectors.

*NOTE:* All experiments involving live animals, including the isolation of primary cells, must be reviewed and approved by the relevant animal care and use committee and must conform to government regulations for the care and use of laboratory animals.

### Design of Lipstic-Engineered Ligand and Receptor Pairs

The initial step to take advantage of the LIPSTIC approach to track cell-cell interactions is to select a ligand and receptor pair and to design their modification with G5 and SrtA tags. The selection of a ligand and receptor pair depends on the biological question that will be addressed; nevertheless, there are a few technical aspects that should be taken into account. First, the G5 tag must be present at the N-terminus of the molecule of interest; thus type II membrane proteins (with the C-terminus present at their extracellular portion) are not possible candidates for this modification. Second, SrtA fusion (which in contrast with the G5 tag can be both at the C- or N-terminus of the molecule of interest) might interfere with protein folding. Even if we successfully fused SrtA to several immune receptors with different structures, favoring single-pass monomeric receptors for SrtA modification might increase the possibility of success.

Once a ligand and receptor pair has been selected, we propose the following workflow to design LIPSTIC-engineered molecules. As an example, we perform the workflow for the murine interacting molecules CD40 and CD40L.

**Figure 2:**
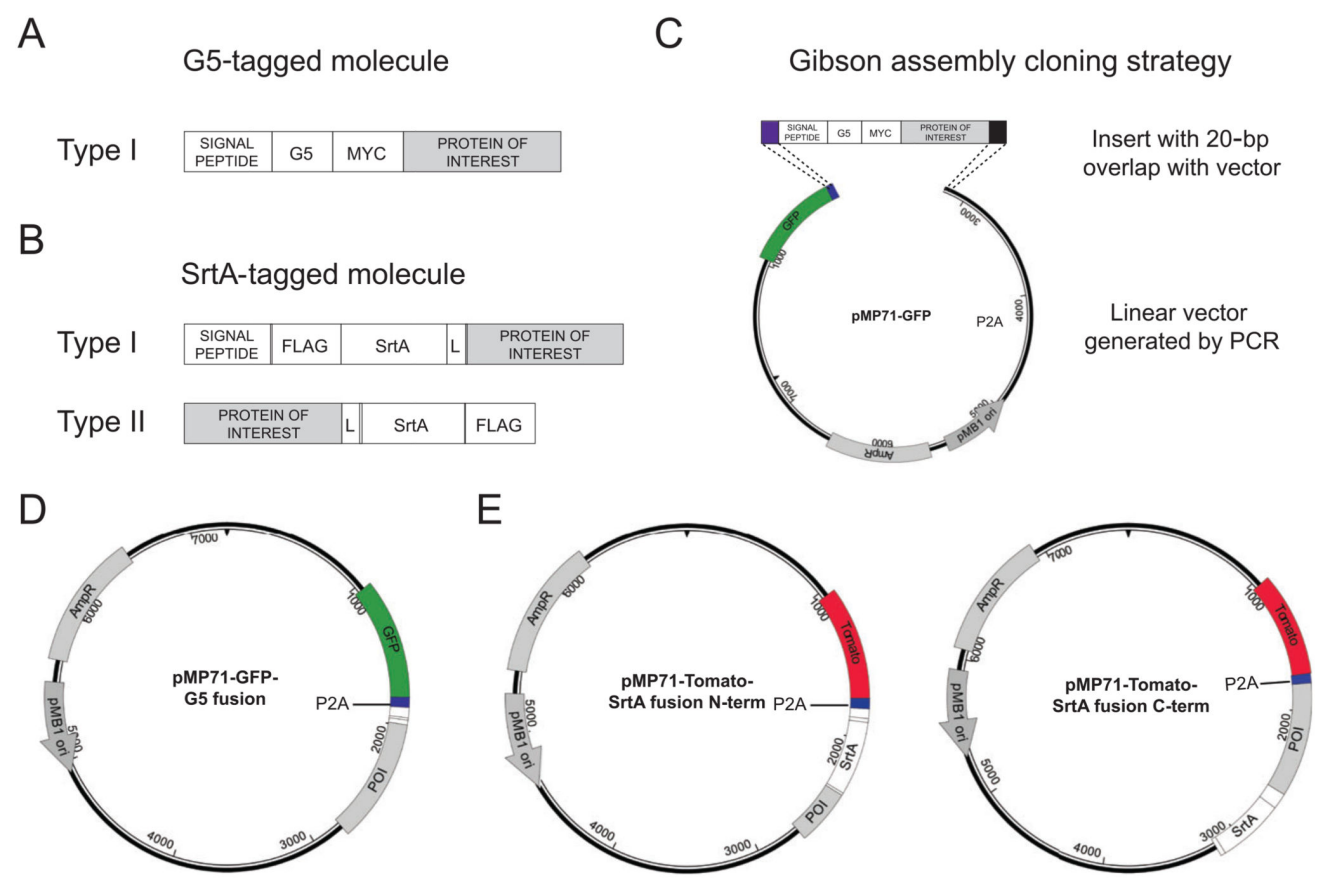
Schematic representation of LIPSTIC-engineered molecules and cloning vectors. (**A**) Five glycine (G5)-tagged molecules are designed to carry a five glycine tag at their extracellular N-terminal portion. Based on this requirement, only type I membrane proteins (i.e., proteins with the N-terminus exposed on their extracellular moiety) are eligible for this modification. The coding sequence of the G5 tag should be preceded by the coding sequence of a signal peptide to allow correct membrane topology and followed by the coding sequence of Myc tag to enable construct detection. (**B**) Sortase A (SrtA)-tagged molecules are designed to carry SrtA at their extracellular portion. Depending on the topology of the molecule to be engineered, SrtA can be added at the N-terminus (type I proteins) or at the C-terminus (type II proteins). In the first case, the coding sequence of SrtA should be proceeded by the coding sequence of a signal peptide and a FLAG tag and should be followed by a linker. In the second case, the protein of interest will be genetically fused to a linker, SrtA, and FLAG tag. (**C**) Schematic representation of Gibson assembly cloning strategy. DNA inserts are modified to carry at both ends a 20-bp overlap with the receiving vector. The receiving linear vector is generated by PCR amplification.Combination of insert and vector with Gibson assembly enzyme mix will lead to the generation a circular plasmid. (**D** and **E**) Schematic representation of retroviral vectors allowing the expression of G5 (D) and SrtA (E) fusion constructs. In all three vectors, the coding sequence of the LIPSTIC-engineered protein of interest is preceded by the coding sequence of a fluorescent protein (eGFP or Tomato) and of the P2A peptide, which thanks to ribosome skipping allows for the expression of two distinct protein products from a single transcript.

1. Identify the correct gene symbol for the ligand and receptor of interest at https://www.genenames.org.
  *Other databases can be used for this purpose. The correct gene symbols for our example are CD40 and CD40LG*.
2. Identify and download the coding nucleotide sequence of the gene of interest at *https://www.ncbi.nlm.nih.gov* by browsing the nucleotide database.
  *It is necessary to specify the correct gene symbol and species*.
  *The correct nucleotide coding sequences corresponding to CD40 and CD40LG genes in Mus musculus are identified by accession numbers M83312.1 and NM_011616.2, respectively. We recommend the use of molecular biology software for the annotation and manipulation of nucleic acid and protein sequences. We currently employ SeqBuilderPro (Lasergene), but equivalent alternatives are available (e.g., SnapGene)*.
3. If not known, predict the membrane topology of the protein of interest and, if present, the cleavage site of the signal peptide at *https://services.healthtech.dtu.dk/service.php?SignalP-6.0*.
  *For CD40, cleavage of the signal peptide occurs between C in position 23 and V in position 24. Results of CD40 sequence analysis provided by the SignalP platform are shown in* Supplementary Figure 1.
4. For the engineering of G5-tagged construct, insert the coding sequence of G5 residues and of the Myc tag immediately after the signal peptide cleavage site (Fig. 2A).
  *The insertion of a Myc tag will facilitate detection of the engineered construct*.
  *G5, Myc tag, and the correctly assembled G5-Myc-CD40 coding sequences are available in* Supplementary Table 1.
5. For the engineering of SrtA fusion construct in type I membrane proteins, insert the coding sequences of FLAG tag, SrtA, and a linker immediately after the signal peptide cleavage site. For the engineering the SrtA fusion construct in type II membrane proteins, insert the coding sequence of a linker, SrtA, and FLAG tag immediately before the STOP codon of the protein of interest (Fig. 2B).
  *The insertion of a FLAG tag will facilitate detection of the engineered construct, while the linker between the protein of interest and SrtA will facilitate correct folding*.
  *FLAG tag, SrtA, linker, and the correctly assembled CD40L-linker-SrtA-FLAG coding sequences are available in* Supplementary Table 1.
  *At the end of this protocol, the investigator should have assembled the complete coding sequences of the G5- and SrtA-tagged molecules. We recommend verifying that all coding sequences are assembled in frame and contain a STOP codon at the 3′ end*.

### Cloning of Lipstic-Engineered Ligand and Receptor Pairs

Once the design of LIPSTIC-engineered molecules has been completed according to Basic Protocol 1, it is necessary to clone the coding sequences of interest into expression vectors. We use retroviral vectors derived from pMP71 (Engels et al., 2003), which were initially developed to achieve high transduction efficiency and robust transgene expression in T cells. In our hands these vectors perform very well for the transduction of primary mouse B and CD4^+^ T lymphocytes but can also be employed for the transfection of other cell types. To clone the designed sequences into pMP71 vectors, we recommend employing a Gibson assembly cloning strategy (Gibson et al., 2009). With this approach, it is sufficient to generate DNA fragments with 20-bp overlaps with the vector and to incubate them in the presence of a combination of 5′ exonuclease, DNA polymerase, and DNA ligase to obtain the assembled DNA product (Fig. 2C). Importantly, we modified pMP71 vectors to express a fluorescent protein followed by the P2A peptide, which induces ribosome skipping during translation (Kim et al., 2011). We refer to these vectors as pMP71-GFP-P2A and pMP71-Tomato-P2A. Insertion of the desired construct after the P2A sequence allows the expression of two distinct protein products (in our case the fluorescent reporter and the LIPSTIC-engineered molecule) from the same transcript (Fig. 2D,E). Here we describe the step-by-step procedure to generate pMP71 vectors and LIPSTIC inserts with a 20-bp overlap and to rapidly assemble them using Gibson assembly.

#### Materials

LIPSTIC-engineered molecule (see Basic Protocol 1)
pMP71 vector encoding GFP-P2A or Tomato-P2A coding sequences
10 mM dNTP mix
Primers to amplify pMP71 vectors:
  Forward F1: AATTCGAGCATCTTACCGCC
  Reverse R1: CGGTCCAGGGTTCTCCTCCA
LongAmp^^®^^ Taq DNA Polymerase (e.g., New England BioLabs, cat. no. M0323) or other high-fidelity polymerase suitable for long-range amplification
Nuclease-free water
0.8% TAE agarose gel
GeneJET Gel Extraction Kit (e.g., Thermo Fisher Scientific, cat. no. K0691) or similar
Gibson Assembly Master Mix (e.g., New England BioLabs, cat. no. E2611S)
Stbl3 chemically competent *Escherichia coli* (e.g., Thermo Fisher Scientific, cat. no. C737303)
SOC medium
LB agar plates with 100 μg/ml ampicillin
LB liquid medium with 100 μg/ml ampicillin
QIAprep Spin Miniprep Kit or other similar plasmid DNA purification kit
Primer to sequence cloned insert:
  Forward F2: CGGAGCTACTAACTTCAGCC
  Reverse R2: ATGGGAATAAATGGCGGTAAGAT

PCR tubes
Thermal cycler
Microvolume spectrophotometer
Static incubator
Orbital shaker for bacterial liquid culture
42°C water bath

Additional reagents and equipment for agarose gel electrophoresis (see *Current Protocols* article: Voytas, 2001)

#### PCR amplification and Gibson assembly

1. Add the following sequences to the coding sequences of LIPSTIC-engineered molecules:
  At the 5′ end: TGGAGGAGAACCCTGGACCG
  At the 3′ end: AATTCGAGCATCTTACCGCC.
  *This will ensure 20-bp overlap with the vector sequence. These sequences are identical for both receiving vectors pMP71-GFP-P2A and pMP71-Tomato-P2A*.
2. Order synthetic genes of the LIPSTIC-engineered molecules including the 20-bp overlap sequences specified in step 1.
  *We routinely order synthetic genes from Integrated DNA Technology, but there are several synthetic gene providers available around the world*.
3. Amplify pMP71 vector using primers F1 and R1.
  *Vectors are available upon request to the corresponding author. The F1 primer anneals after the 3′ end of the insert insertion site, and the R1 primer anneals at the 3′ end of the P2A sequence*.
  - Set up the reaction in PCR tubes as follows:
    1 ng template DNA
    1.5 μl of 10 mM dNTPS
    2 μl of 10 μM primer F1
    2 μl of 10 μM primer R1
    10 μl of 5× buffer (provided with LongAmp^®^ Taq DNA polymerase)
    2 μl LongAmp^^®^^ Taq DNA polymerase
    Nuclease-free water to 50 μl.
  - Run PCR in a thermal cycler using the following cycle conditions:

**Table:** 

Initial denaturation	94°C	30 s
30 cycles	94°C	30 s
	55°C	60 s
	65°C	5:50 min
Final extension	65°C	10 min
Hold	4°C to 10°C	–
4. Run PCR product on a 0.8% TAE agarose gel. Cut band and purify DNA using GeneJET Gel Extraction Kit according to the manufacturer’s instructions.
  *The expected amplicon size is 6262 bp for pMP71-Tomato-P2A vector and 6335 bp for pMP71-GFP-P2A vector*.
5. Quantify concentration of the purified DNA.
6. Perform Gibson assembly.
  - Set up the reaction as follows:
    100 ng vector DNA (from step 4)
    3-fold molar excess (vs vector) insert DNA (from step 2)
    10 μl Gibson assembly master mix
    Nuclease-free water to 20 μl.
  - Incubate reaction at 50°C for 15 min, and then store on ice or at –20°C until step 7.
    *We recommend cloning G5- and SrtA-tagged molecules in receiving vectors expressing different fluorescent reporters to easily distinguish differently functionalized cells in coculture experiments*.

#### Bacterial transformation

7. Thaw chemically competent *E. coli* on ice.
  *We recommend using the Stbl3 strain, which has been optimized for cloning and propagating lentiviral and retroviral vectors*.
8. Add 4 μl Gibson assembly reaction to a 20 μl bacteria suspension, and incubate on ice for 5 min.
9. Heat shock bacteria for 30 s at 42°C by placing bacteria in a water bath.
10. Place bacteria on ice for 5 min.
11. Add 200 μl SOC medium, and shake bacteria at 37°C for 1 hr.
12. Plate bacteria on LB ampicillin plate, and incubate at 37°C overnight.

#### Confirm transformants

13. Pick four colonies, and inoculate 3 ml LB ampicillin for each clone.
14. Incubate liquid culture on a shaker at 37°C overnight.
15. Harvest liquid cultures, and isolate plasmid DNA with QIAprep Spin Miniprep Kit according to the manufacturer’s instructions.
16. Perform Sanger sequencing of plasmid DNA using primers F2 and R2. If needed, design and use additional sequencing primers specific for the insert sequence.
  *Primer F2 anneals at the 3′ end of the P2A sequence, and primer R2 anneals after the 3′ end of the insert*.
  *At the end of this protocol, the investigator should have generated expression vectors encoding the G5- and SrtA-tagged molecules of interest. We recommend carefully verifying the entire sequence of the insert before proceeding with Basic Protocol 3*.

### Validation of Lipstic-Engineered Ligand and Receptor Pairs in 293T Cells

**Figure 3:**
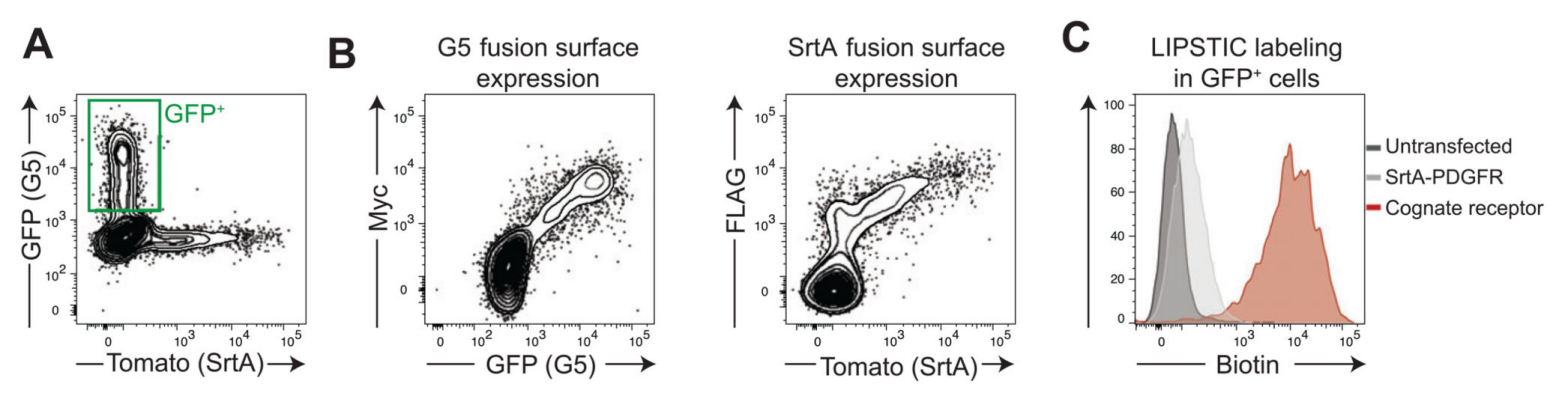
Validation of LIPSTIC-engineered receptor in vitro. 293T cells were transfected with G5-CD40 (GFP reporter vector), CD40L-SrtA (Tomato reporter vector), or SrtA-PDGFR (Tomato reporter vector) or were left untransfected. At 48 hr post transfection, cells were mixed and incubated at room temperature in the presence of 100 μM biotin-LPETG. Cells were washed, stained, and analyzed by fluorescence-activated cell sorting. (**A**) Identification of GFP^+^ cells transfected with a GFP reporter vector and Sortase A (SrtA)^+^cells transfected with a Tomato reporter vector. (**B**) Verification of cell surface expression of five glycine (G5) and SrtA fusion constructs based on Myc and FLAG tag, respectively, staining. (**C**) LIPSTIC enzymatic labeling on G5-expressing cells, gated as in A.

Once LIPSTIC-engineered molecules have been cloned into expression vectors, it is necessary to assess a few parameters to validate the novel constructs: (1) cell surface expression; (2) presence of enzymatic activity in SrtA fusion constructs; and (3) interaction-specific LIPSTIC labeling. To evaluate these aspects, we suggest individually transfecting 293T cells with LIPSTIC-engineered ligand and receptors, performing LIPSTIC labeling in vitro, and analyzing cells by flow cytometry. We also recommend including in the assay a control construct, where no interactions are expected, encoding for SrtA fused only to a synthetic transmembrane domain. Plasmid pMP71-Tomato-P2A-SrtA-PDGFR is available from the corresponding author upon request. This protocol informs the investigator on surface expression and activity of the LIPSTIC-engineered constructs and represents the first validation step that should be performed in the development of novel LIPSTIC-engineered ligand and receptor pairs. Representative results of intercellular labeling obtained in 293T cells are shown in Figure 3 and are discussed in the Commentary, Understanding Results.

#### Materials

293T cell line (e.g., ATCC, cat. no. CRL-3216; RRID:CVCL_0063)
Complete Dulbecco’s modified Eagle medium (DMEM; see recipe)
Calcium Phosphate Transfection Kit (e.g., Thermo Fisher Scientific, cat. no. K278001)
Plasmids encoding LIPSTIC-engineered ligand and receptor (see Basic Protocol 2)
Control construct pMP71-Tomato-P2A-SrtA-PDGFR
Phosphate-buffered saline (PBS)
20 mM biotin-LPETG (see recipe)
PBE buffer: PBS containing 0.5% (w/v) bovine serum albumin (BSA) and 2 mM EDTA
Antibodies and reagents for flow cytometry:
  Anti-biotin-APC (e.g., Miltenyi Biotec, cat. no. 130-113-288)
  Anti-Myc-PE-Cy7 (e.g., Novus Biologicals, cat. no. NB600-302PECY7)
  Anti-FLAG-BV421 (e.g., Biolegend, cat. no. 637322)
Formaldehyde

10-cm dishes, cell culture treated
37°C, 5% CO_2_ incubator for mammalian cells
Cell counter
1.5-ml microcentrifuge tubes
Centrifuge
Fluorescence-activated cell sorting (FACS) tubes
FACS analyzer

1. Seed 2 × 10^6^ 293T cells in 10-cm dishes. Prepare four dishes, and culture in complete DMEM.
2. The following day, transfect 293T cells with calcium phosphate according to the manufacturer’s instructions. Include the following constructs:
  pMP71-GFP-P2A-G5 fusion
  pMP71-Tomato-P2A-SrtA fusion
  pMP71-Tomato-P2A-SrtA-PDGFR.
  *Alternatively reagents can be prepared in house according to a Current Protocols article by Kingston, Chen, & Rose (2003)*.
3. Leave one dish untransfected.
  *Untransfected cells will be used as a negative control in the experimental setup and are required to properly set gates (transfected/untransfected) in the following FACS analysis*.
4. Two days post transfection, detach 293T cells from dishes, and wash with PBS. Resuspend cells in PBS at 10^7^ cells/ml. Distribute 100 μl/tube G5-transfected cells in three 1.5-ml tubes. Add 100 μl untransfected cells, pMP71-Tomato-P2A-SrtA fusion, or pMP71- Tomato-P2A-SrtA-PDGFR transfected cells.
5. Add SrtA substrate biotin-LPETG to a final concentration of 100 μM.
  *Aliquots of biotin-LPETG stock solution can be stored at –20°C; avoid freeze-thaw cycles*.
6. Incubate cells in the presence of substrate for 30 min at room temperature.
7. Centrifuge cells 5 min at 300 × *g*, 4°C. Remove supernatant and wash cells by adding 1.5 ml cold PBE.
  *The washing step is extremely important since it allows for removing excess biotinylated substrate that, if not properly removed, will quench the anti-biotin antibody used for FACS staining*.
8. Centrifuge cells 5 min at 300 × *g*, 4°C. Remove supernatant and resuspend cells in 100 PBE. Add antibodies for FACS staining with the following dilutions: anti-biotin-APC (1:50), anti-Myc-PE-Cy7 (1:400), and anti-FLAG-BV421 (1:400).
  *We suggest this combination of fluorophores, which will perform well in multiple instruments; nevertheless, the combination of fluorophores can be adjusted by the investigator based on the setup of available instruments*.
9. Incubate samples 15 min at 4°C.
10. Wash samples with 1 ml PBE. Resuspend in 200 μl PBS containing 2% (w/v) formaldehyde, and transfer to FACS tubes.
11. Analyze samples by flow cytometry.
  *Representative data are shown in* Figure 3.
  *We currently employ BD LSR-II and Fortessa equipped with four lasers (405, 488, 561, and 633 nm). Other instruments can be used to perform the analysis, with a minimum requirement of the ability to measure six fluorescent parameters*.
  *Compensation setup of the instrument can be performed with compensation beads (e.g., Thermo Fisher Scientific, cat. no. 01-1111-41), untransfected unstained cells, transfected unstained cells, and untransfected single-stained cells*.

### Measuring Interaction With Lipstic in Immune Cells Ex Vivo

**Figure 4:**
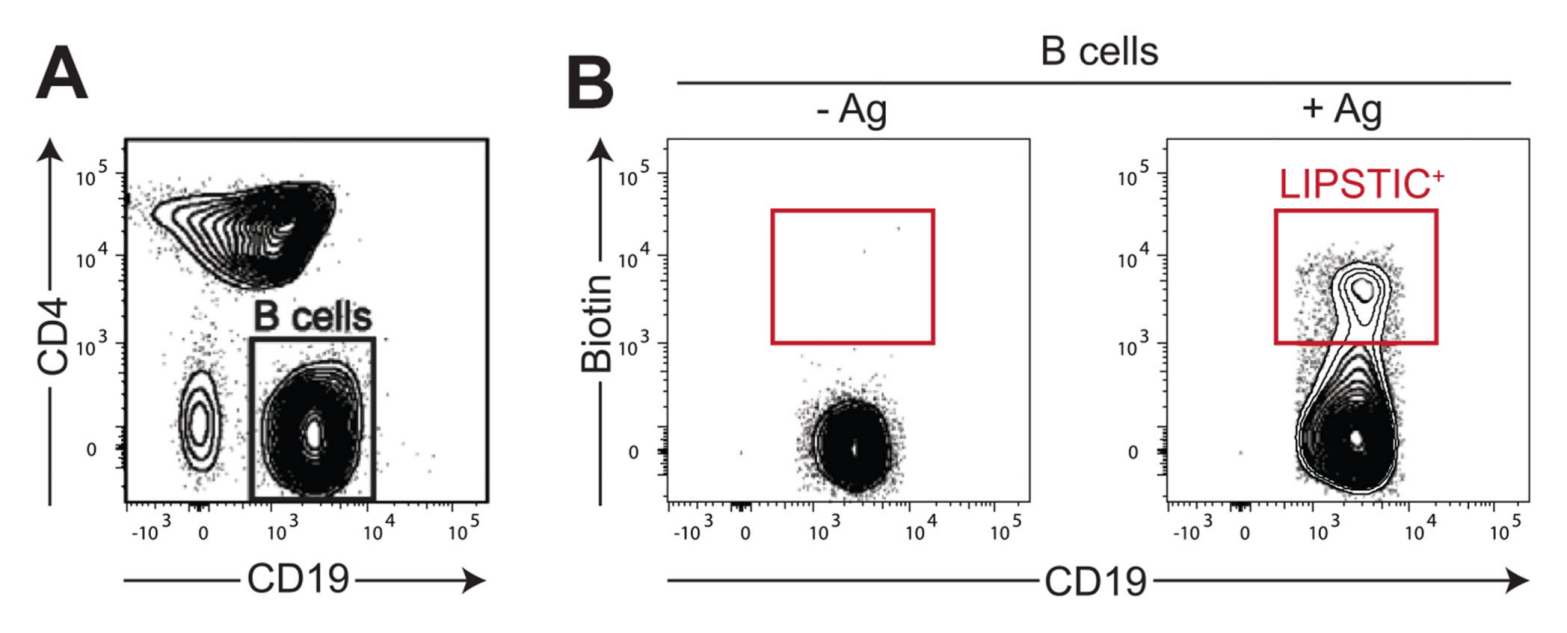
Measuring interaction with LIPSTIC in immune cells ex vivo. B cells isolated from *Cd40^G5/G5^* mice were co-cultured with CD4^+^ T cells isolated from *Cd40lg*^SrtA/Y^ CD4-Cre OT-II animals in the presence or absence of the cognate antigen (Ag). After 6 hr, cells were treated with biotinylated SrtA substrate for 30 min and then analyzed by flow cytometry. (**A**) Identification of B and T cells in the co-culture based on expression of CD19 and CD4 markers. (**B**) LIPSTIC labeling identified by biotin cell surface staining in CD19^+^ cells gated as in A.

Once surface expression and functionality of LIPSTIC-engineered molecules have been initially assessed in 293T cells, it is advisable to further characterize the constructs in the context of the target immune cells. Assays with this aim will strongly depend on the molecules involved and on their biological function, and thus it is not possible to provide a one-size-fits-all protocol to guide the investigator in this task. Nevertheless, once the constructs have been characterized, their use to measure interaction ex vivo can follow a simple strategy that we have successfully employed with murine B cells, T cells, and dendritic cells. As a reference, here we provide details on how to measure interactions between primary murine B and T cells. This experimental setup can be exploited to rapidly investigate how the perturbation of choice (e.g., genetic modifications, pharmacological treatments, antigen recognition, nature of the immune stimulation) affects immune interactions. Moreover, cells undergoing interactions can be analyzed pheno-typically (e.g., by characterizing cell surface marker expression by flow cytometry) or isolated by FACS sorting for downstream analysis. Representative results are shown in Figure 4 and discussed in the Commentary, Understanding Results.

#### Materials

B and T cells carrying LIPSTIC-engineered molecules
Complete RPMI (see recipe)
20 mM biotin-LPETG (see recipe)
PBE buffer: PBS containing 0.5% (w/v) BSA and 2 mM EDTA
FC block (e.g., BD Biosciences, cat. no. 553141)
Antibodies and reagents for flow cytometry:
  Anti-biotin-APC (e.g., Miltenyi Biotec, cat. no. 130-113-288)
  Anti-CD4-BV421 (e.g., Biolegend, cat. no. 100437)
  Anti-CD19-PE-Cy7 (e.g., Biolegend, cat. no. 115520)

96-well U-bottom plates
37°C, 5% CO_2_ incubator for mammalian cells
Refrigerated centrifuge with swinging bucket rotor and plate adaptor
FACS tubes
FACS analyzer

1. Distribute 2 × 10^5^ cells/well LIPSTIC-engineered B and T cells in a 96-well U-bottom plate in a final volume of 200 μl complete RPMI.
  *LIPSTIC-engineered cells can be obtained by retroviral transduction of primary murine B and T lymphocytes, by CRISPR/Cas9 genome editing, or by isolation from LIPSTIC-engineered mouse strains as in the case of Cd40^G5^ and Cd40lg^SrtA^ animals. Detailed protocols for all these procedures have been previously published (see Current Protocols article: Huang, Johansen, & Schwartzberg, 2019; Lee, Sadelain, & Brentjens, 2009; Pasqual, Chudnovskiy, & Victora, 2021). The investigator should be aware that the retroviral constructs described in Basic Protocol 1 are ideal for retroviral transduction of primary murine B and T lymphocytes, allowing for high transduction rates*.
  *We recommend a 1:1 ratio between B and T cells*.
  *Seeding cells on a 96-well U-bottom plate will favor the concentration of cells on a small surface. Despite the high density reached by the cells in this setup, we observed that labeling specificity is not affected. Alternatively, 96-well flat-bottom plates or other larger formats can be employed if a larger number of cells are available for testing*.
2. At the desired time point, add 20 μl/well of 1.1 mM biotin-LPETG solution in RPMI to a final concentration of 100 μM/well.
  *If working in this format, it is not necessary to mix or resuspend the cells upon addition of the substrate*.
  *The choice of timing depends on the kinetics of the interaction, which will be monitored. As a reference, when tracking the CD40-CD40L interaction, we co-culture cells for a minimum of 6 hr to a maximum of 24 hr before adding SrtA substrate*.
3. To allow for the SrtA labeling reaction, incubate co-culture in the presence of the substrate for 30 min at 37°C.
4. Wash cells three times with cold PBE to remove excess substrate. Perform washing as follows: Centrifuge plate 5 min at 300 × *g*, 4°C, and discard supernatant by firmly inverting the plate. Then resuspend cells in 200 μl PBE.
  *Washing and subsequent FACS staining can be performed directly in the 96-well plate as detailed. This approach is convenient when a large number of conditions are being analyzed. Alternatively, when the SrtA labeling reaction is terminated, cells can be transferred to tubes and washed and stained in this format*.
5. After the last wash, resuspend cells in 50 μl/well PBE containing 2 μg/ml Fc block. Incubate at room temperature for 5 min.
6. Add 50 μl/well of 2× antibody staining mix.
  *The final dilutions of the antibody are as follows: 1:50 anti-biotin-APC, 1:400 anti-CD4-BV421, and 1:400 anti-CD19-PE-Cy7*.
7. Incubate sample 15 min at 4°C.
8. Wash cells two times with cold PBE, and resuspend cells in 200 μl PBE.
  *If the cells are not analyzed the same day, the samples can be fixed in 2% (w/v) formaldehyde. Fixed samples are stable for at least 1 week if stored at 4°C*.
9. Analyze samples by flow cytometry.

### Reagents and Solutions

#### Biotin-LPETG, 20 mM

20 mM biotin LPETG (lyophilized)
PBS
For long-term storage, store peptide at –80°C
Store resuspended aliquots at –20°C for up to 1 month
  *The biotinylated peptide can be purchased as custom synthesis from various companies. We routinely order it from Lifetein and Genscript specifying the following information: peptide sequence = SELPETG; N-terminal modification = biotin-aminohexanoic acid; C-terminal modification = amidation; and purity = 98%*.

#### Complete DMEM

DMEM with glutamine
10% (v/v) heat-inactivated fetal bovine serum
100 U/ml penicillin, 100 μg/ml streptomycin
Store at 4°C for up to 3 months

#### Complete RPMI

RPMI with glutamine
10% (v/v) heat-inactivated fetal bovine serum
100 U/ml penicillin, 100 μg/ml streptomycin
Store at 4°C for up to 3 months

### Commentary

#### Background Information

LIPSTIC technology allows for the enzymatic labeling of cells engaged in specific contact-dependent interaction, either in vitro or in vivo, in the living mouse. This technology has been developed with a clear goal: to make cell-cell interactions easily measurable and interaction history finally visible. Technically, LIPSTIC relies on the genetic engineering of ligand and receptor pairs involved in interaction by the addition at their extracellular portion of either the enzyme SrtA or G5 tag. Upon interaction, SrtA can catalyze the transfer of a biotinylated or fluorescently labeled substrate to the G5 tag, so that the cell that underwent interaction will display a detectable label on its surface.

Before the development of LIPSTIC, SrtA has been widely exploited as a tool to achieve site-specific protein modifications. Its broad applications in vitro include protein cyclization (Popp, Dougan, Chuang, Spooner, & Ploegh, 2011), modification of proteins displayed on the surface of living cells (Popp, Karssemeijer, & Ploegh, 2012; Shi et al., 2014), and conjugation to purified proteins of virtually any sort of moiety—dyes (Popp, Antos, Grotenbreg, Spooner, & Ploegh, 2007), lipids (Antos, Miller, Grotenbreg, & Ploegh, 2008), nucleic acids (Pritz et al., 2007), and drugs (Beerli, Hell, Merkel, & Grawunder, 2015), just to mention a few examples. The SrtA target motif has also been inserted into genetically engineered mice to generate SrtA-ready surface receptors (Chen et al., 2017; Shi et al., 2014) and antibodies (Maruyama et al., 2016) for ex vivo modification. In vivo SrtA activity has been employed to catalyze surface modification (Ham et al., 2016). Our choice of using SrtA to achieve intercellular labeling was primarily motivated by three aspects. First, SrtA is relatively small in size, at ~25 kDa. We have observed experimentally that its fusion to cellular receptors is better tolerated than that of bulkier enzymes. Second, several SrtA variants with different kinetic parameters have been engineered (Chen, Dorr, & Liu, 2011), giving us the possibility to easily modulate enzyme activity based on our needs. Third, SrtA substrate is biocompatible and can tolerate a large variety of modifications, thus allowing large flexibility in terms of detection system. Together, these features prompted us to use SrtA to set up a novel tool to label cell-cell interactions; nevertheless, given the interest in the field of protein engineering toward the development of novel strategies for protein modification, we anticipate that an increasing number of enzymes with similar characteristics will be available in the near future.

##### Comparison with other methods

Cell-cell interactions have long been investigated exclusively by imaging approaches. Thanks to these approaches, it is possible to visualize cell-cell contacts and to characterize multiple dynamic aspects of cell-cell interactions such as duration and frequency. When performed in vivo, live-imaging approaches can also reveal the microanatomical localization where interactions take place. Nevertheless, in this experimental setup, cells undergoing interactions cannot be identified and retrieved for downstream analysis, and therefore the phenotypic and molecular changes associated with the interaction cannot be investigated.

In contrast with imaging, LIPSTIC offers the major advantage of marking the cells undergoing interaction in vitro and in vivo in a way that they can be later identified and isolated for downstream applications such as RNA sequencing and functional assays. Moreover, if a fluorescently labeled SrtA substrate is employed, LIPSTIC labeling can also be combined with live-imaging approaches to benefit from the advantages of both techniques in a single experiment.

An emerging approach to monitoring cell-cell interactions is histocytometry, a high-content imaging approach that combines positional information with in-depth phenotypic characterization of cells by staining with multiple fluorescent probes (Gerner, Kasten-muller, Ifrim, Kabat, & Germain, 2012). Given its high resolution, this approach is also able to infer cell-cell communication based on proximity patterns, thus providing an anatomical description of the environment within which cell-cell interactions occur. We anticipate that this approach could be combined with the detection of LIPSTIC labeling to obtain a phenotypic characterization of interacting cells in situ. Collectively, the combination these different experimental techniques will provide unprecedented resolution to studies of the dynamics of the immune response and, in general, of cell-cell interactions.

##### Applications of LIPSTIC

**Figure 5:**
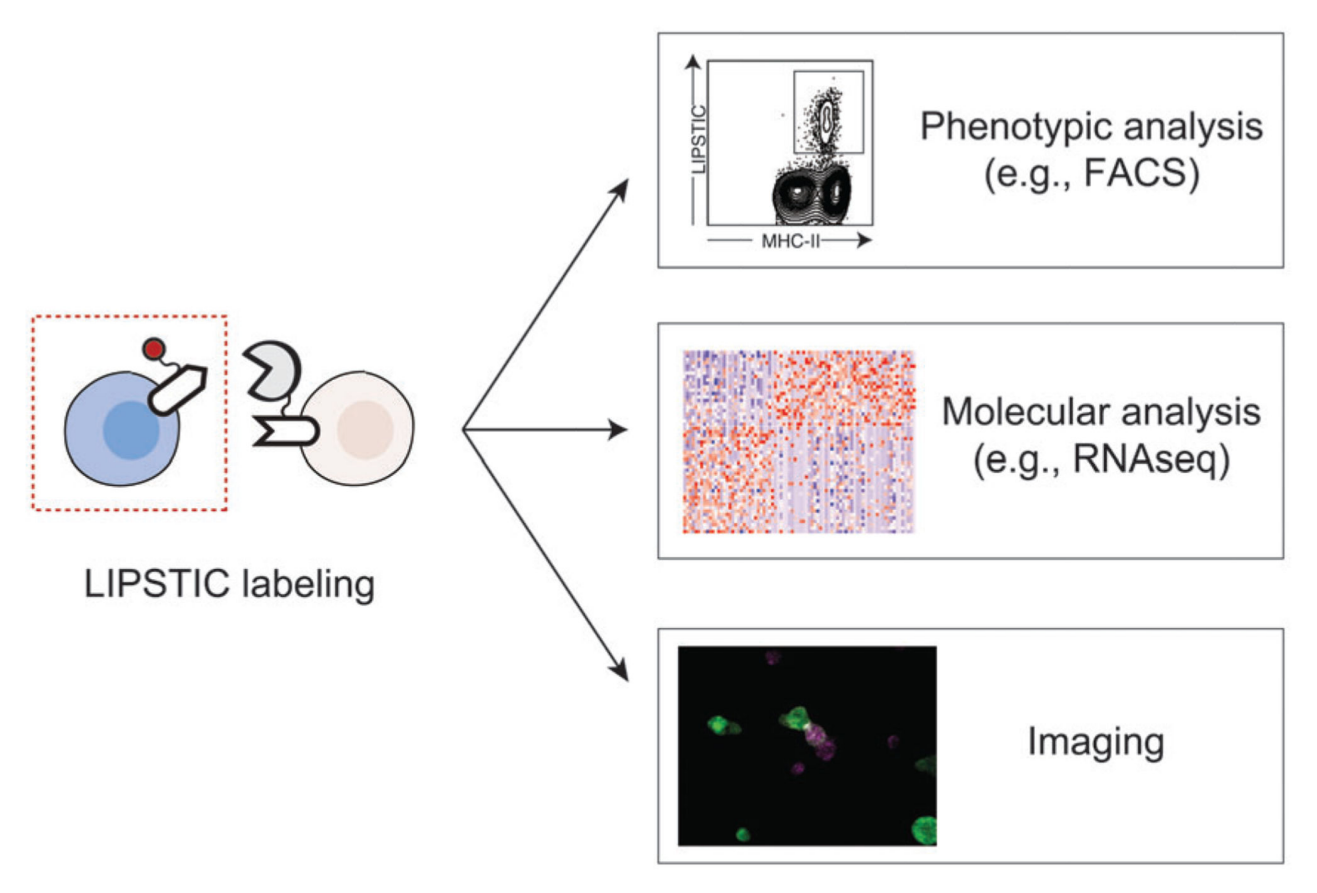
Potential application of LIPSTIC. LIPSTIC labeling can be performed in vitro or in vivo. Labeled cells can be analyzed phenotypically by fluorescence-activated cell sorting (FACS), sorted and analyzed by molecular approaches such as RNA sequencing (RNAseq), or visualized by imaging.

We have so far used LIPSTIC ex vivo and in vivo to track CD40-CD40L interactions, but the same approach could be extended to other ligand-receptor pairs. As proof of principle, we presented data showing that the LIPSTIC tagging strategy can be applied to several molecules including CD28/CTLA4-CD80/CD86, PD1-PDL1/PDL2, and ICOS-ICOSL (Pasqual et al., 2018). We envision that combining LIPSTIC with transcriptional profiling of interacting cells and functional assays will enable us to characterize the effects of the signals exchanged upon cell-cell communication mediated by each of these molecular partnerships. Finally, although our primary goal was to develop technology to study cell-cell interactions in vivo, LIPSTIC could have several uses in vitro (e.g., as a screening platform to identify interacting molecules displayed on the cell surface; Fig. 5).

##### Limitations of LIPSTIC

LIPSTIC requires genetic modification of a ligand-receptor pair involved in the cell-cell interaction of interest. Thus, interactions involving unknown receptor-ligand pairs cannot at present be monitored using this system. Moreover, for in vivo applications, this implies the generation of two genetically modified mouse lines per LIPSTIC pair, which is time consuming. The implementation of CRISPR/Cas9-based genome editing tools in mouse zygotes has drastically shortened the time required for the generation of novel mouse strains (Maruyama et al., 2016; Quadros et al., 2017; Wang et al., 2013), thus mitigating this limitation.

A second limitation is that modification of ligands and receptors by fusion of SrtA (and, less likely, of the G5 tag) can affect signaling properties, and thus the functionality of every novel LIPSTIC ligand-receptor pair needs to be assessed experimentally. This is an obvious issue when the function of that particular receptor-ligand pair is important for the interaction under scrutiny but may be less of a problem when LIPSTIC is used only to identify partner cells able to undergo a given interaction (e.g., finding which subset of dendritic cells are presenting antigen to T cells under different conditions).

#### Critical Parameters

As any enzymatic reaction, the efficiency and specificity of LIPSTIC labeling is affected by several variables that should be taken into account when designing LIPSTIC-based approaches.

##### Affinity of SrtA for G5 tag

To be able to selectively obtain SrtA intercellular labeling upon a specific cell-cell interaction, we employed in LIPSTIC an engineered variant of the SrtA enzyme (SrtA P94S/D160N/K196T) that displays lower affinity toward the G5 tag (*K*_m_ = 1830 μM) than the wild-type counterpart (Chen et al., 2011). Since the G5 tag is poorly recognized by this variant, the intercellular labeling reaction is unlikely to be driven by the affinity of SrtA to G5 but rather occurs upon cell-cell interaction as a consequence of increased local concentration of the tagged ligand and receptor.

##### SrtA and G5 tag expression levels

Since ligand-receptor interactions are strongly influenced by their expression level, we generated mice carrying LIPSTIC-engineered ligand and receptor inserted into the endogenous loci to maintain endogenous transcriptional regulation and levels of expression. This approach ensures that the intercellular labeling observed experimentally reflects the biological interaction that is being assessed. However, one can easily envision other scenarios where synthetic transcriptional regulation of SrtA- and G5-tagged molecules may be a valuable alternative. For instance, since SrtA and G5 expression levels directly influence the rate of product formation, very low expression levels might result in the selective labeling of long-lived interactions. Thus, depending on the specific biological question that needs to be addressed, the levels of SrtA and G5 expression and their regulation can be experimentally manipulated.

##### SrtA substrate concentration and reaction times

The SrtA variant in use in our LIPSTIC system has a *K*_m_ of 560 μM toward the LPETG motif (Chen et al., 2011). We routinely use in vitro and ex vivo SrtA substrate concentrations ranging from 10 to 100 μM and reaction times of 20 to 30 min. In vivo, different doses of substrates, depending on the route of administration and target anatomical site, are injected over a 2-hr period. These conditions result in robust intercellular labeling that is dependent on ligand-receptor interaction. We anticipate that both reaction time and substrate concentration will require experimental testing in novel ligand/receptor LIPSTIC pairs.

#### Troubleshooting

Table 1 provides a list of possible problems and solutions that scientists might experience performing the protocols described.

#### Understanding Results

Once cloning and design of LIPSTIC-engineered constructs has been accomplished, it essential to check both the functionality of the constructs and the specificity of LIPSTIC labeling. We propose initially validating these aspects in 293T cells by simultaneously testing (1) cell surface expression of the engineered constructs by detection of Myc and FLAG tags and (2) LIPSTIC intercellular labeling between G5- and SrtA-expressing cells. We expect to obtain a correlation between Myc and GFP signals (G5 constructs) and between FLAG and Tomato signals (SrtA constructs), as exemplified in Figure 3B. Moreover, we expect to observe specific LIPSTIC labeling on the surface of G5-expressing cells when a specific interaction is occurring between G5- and SrtA-expressing cells. In other words, given cells expressing a G5-tagged receptor incubated with cells expressing a SrtA-tagged ligand, we expect a significant shift in biotin signal only when SrtA is fused with the cognate ligand rather than with a noninteracting molecule (e.g., SrtA-PDGFR). This is exemplified in Figure 3C.

Once LIPSTIC-engineered molecules have been validated, they can be employed to measure interactions by different means such as by the generation of knock-in animals carrying the mutations of interest or by the genetic modification of immune cells, in particular lymphocytes, using CRISPR/Cas9 or retroviral transduction. The first option, despite being time consuming, has the great advantage of maintaining endogenous transcriptional regulation of the engineered molecules and minimizing variability across experiments. Other options requiring ex vivo manipulations of immune cells are definitely faster to achieve, but the use of artificial promoters might significantly alter the biology of the molecules affected. Despite these differences in the possible sources of LIPSTIC-engineered cells, in Basic Protocol 4 we provide a general protocol that we successfully employed to measure interactions with LIPSTIC ex vivo with retro-virally transduced lymphocytes and with primary lymphocytes isolated from knock-in animals. In Figure 4 we show the results obtained when B cells isolated from *Cd40*^G5/G5^ mice were co-cultured with CD4^+^ T cells isolated from *Cd40lg*^SrtA/Y^ CD4-Cre OT-II animals in the presence or absence of the cognate antigen. In this setting, after LIPSTIC labeling ex vivo, we can clearly detect a population of biotin^+^ B cells but only when the T cell cognate antigen is present. This result reflects the biology of the CD40-CD40L interaction, which in naive CD4^+^ T cells requires antigen recognition to occur.

#### Time Considerations

The design of LIPSTIC-engineered ligand and receptor pair can be accomplished in 1 day. Cloning of the construct in retroviral vector requires up to 1 week. Validation of LIPSTIC-engineered ligand and receptor in 293T cells can be performed in 4 days. A typical LIPSTIC labeling experiment, provided the cells carrying the engineered construct are available, can be performed in 1 day including analysis with flow cytometry.

## Supplementary Material

Supporting Information

## Figures and Tables

**Table 1:** Troubleshooting Guide for LIPSTIC Labeling

Problem	Possible cause	Solution
No substrate signal on cells transfected/transduced with SrtA fusion receptor	No cell surface expression of SrtA fusion receptor due to folding issues	Check cell surface expression of construct by FACS using an antibody specific for the engineered receptor; if fusion construct is not expressed, expression could be rescued by modifying its properties (e.g., linker length/sequence)
	Unbound SrtA substrate carryover during FACS staining	After incubation of cells with SrtA substrate, wash cells abundantly with PBE to remove unbound substrate, which will quench detection antibody during FACS staining
	Unsuitable SrtA substrate solution (wrong concentration, substrate degradation)	Make fresh SrtA substrate solution; to avoid substrate degradation, keep stock solution at –80°C; include already validated SrtA fusion construct (e.g., CD40L-SrtA) as positive control to confirm substrate is working properly
No substrate signal on cells transfected/transduced with G5 fusion receptor upon LIPSTIC labeling	No cell surface expression of G5 fusion receptor	Check cell surface expression of construct by FACS using an antibody specific for the engineered receptor
	G5 tag not present at N-terminus of fusion construct	Reevaluate construct design to ensure G5 tag was inserted after the signal peptide cutting site
	Loss of affinity between LIPSTIC-engineered ligand and receptor	Usually addition of G5 tag does not alter receptor properties, while SrtA fusion might affect protein folding and cause a decrease in affinity between ligand and receptor; measure binding of soluble form of the ligand to SrtA fusion receptor–expressing cells by FACS

FACS, fluorescence-activated cell sorting; G5, five glycine; SrtA, Sortase A.

## Data Availability

The data, tools, and material (or their source) that support the protocols are available from the corresponding author upon reasonable request.
