# Supplementary figures and images for: Engineering Ligand and Receptor Pairs with LIPSTIC to Track Cell-Cell Interactions
Source: Curr Protoc. Author manuscript; Available in PMC 2022 Oct 15. (PMC7613713; doi:10.1002/cpz1.311)

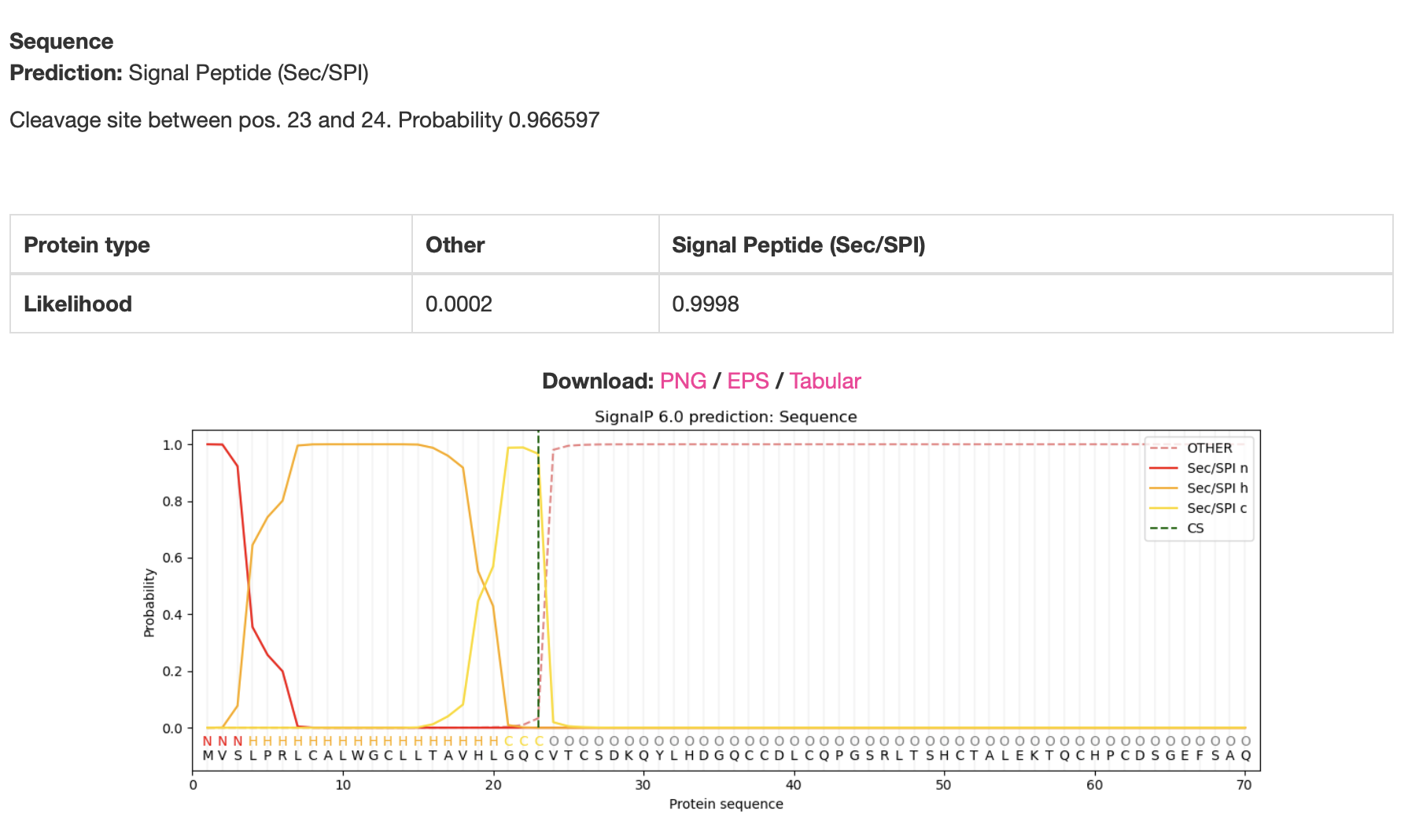

Supplement: Supporting Information [file EMS155368-supplement-Supporting_Information.zip › cpz1311-sup-0001-figures1.png]
